# Complementary feeding practices among infants and young children in Abu Dhabi, United Arab Emirates

**DOI:** 10.1186/s12889-020-09393-y

**Published:** 2020-08-27

**Authors:** Zainab Taha, Malin Garemo, Joy Nanda

**Affiliations:** 1 Department of Health Sciences, CNHS, Zayed University, PO Box 144534, Abu Dhabi, United Arab Emirates; 2 The John Hopkins Medical Institutions, Baltimore, MD USA

**Keywords:** Complementary feeding, Infant nutrition, Toddler, Socio-economics, Abu Dhabi, United Arab Emirates

## Abstract

**Background:**

Optimal complementary feeding (CF) promotes health and supports growth and development in children. While suboptimal feeding practices are reported for many countries, very limited information exists about such practices in the United Arab Emirates (UAE). The present study describes CF practices in Abu Dhabi, UAE, and evaluates them using the United Nations Children’s Fund (UNICEF) Programming Guide: Infant and Young Child Feeding.

**Methods:**

In this cross-sectional study, participating mothers of children below the age of two reported on their children’s CF introduction and practices via a structured questionnaire. The study received ethical approval (ZU17_006_F) from Zayed University.

**Results:**

Out of 1822 participating mothers, 938 had initiated complementary feeding for their children, who had a mean age of 7.1 ± 5.9 months. Three quarters of the children (72.2%) were introduced to CF in a timely manner between the ages of 6 and 9 months. A majority (71.4%) consumed ≥4 food groups, i.e. the recommended minimum diet diversity. In total, less than half (47.3%) of the children met the requirements for minimum meal frequency, with the non-breastfed, 6–23 month old children being the least compliant (21.9%) (*p* < 0.001). Many children were fed with sugar-containing snack items. Overall, 36.2% of the children aged ≥6 months had a minimum acceptable diet.

**Conclusion:**

The gap between the suboptimal CF practices and the recommendations may be attributable to poor knowledge about feeding practices rather than food availability problems. Effective intervention programs can facilitate improvements in the feeding practices to better support a healthy upbringing among Abu Dhabi infants and toddlers.

## Background

Adequate breastfeeding and optimal complementary feeding promote health, support growth and enhance the development of infants [1, 2]. Conversely, suboptimal complementary feeding practices can negatively impact the growth of infants and young children and contribute to health-related problems such as delayed motor and cognitive development, nutrient deficiencies or malnutrition [3].

Complementary feeding is defined as the process starting when breast milk alone is no longer sufficient to meet infants’ nutritional requirements, resulting in the need for other foods and liquids along with breast milk [1, 4]. According to the UNICEF Programming Guide: Infant and Young Child Feeding, appropriate complementary feeding includes timely introduction of complementary feeding, diet diversity and meal frequency [5].

Following the World Health Organization (WHO) recommendations, timely introduction means that complementary feeding should be introduced at 6 months of age. Complementary feeding is needed from that age because breast milk or infant formula alone are not enough to cover the infant’s energy needs or provide sufficient amounts of certain nutrients such as protein, zinc, iron and fat-soluble vitamins [4]. Infants are born with a store of iron in their liver that is sufficient for the first 6 months of life but after that the amount of iron in breast milk will not satisfy infants’ nutritional requirements for iron [6]. In addition to timely introduction, the WHO also emphasizes diet diversity, meaning that a variety of the basic food groups should be included as part of the complementary feeding to ensure a heterogeneous nutrient intake that satisfies all nutrient needs in the growing infant [1].

Like the WHO, the European Society for Pediatric Gastroenterology, Hepatology and Nutrition (ESPGHAN) recommends continued breastfeeding until 2 years of age along with complementary feeding [1, 7]. As infants grow, complementary feeding is needed to bridge the gap in nutrients between infants’ daily requirements and the amount obtained from breast milk or infant formula. Six months is the appropriate age for the introduction of complementary feeding because younger infants are not physiologically ready for anything other than breast milk or infant formula, due to the immaturity of their gastrointestinal system and kidneys [8]. With regard to meal frequency, children may be unable to consume enough food in just a few meals, due to their limited gastric capacity. Hence, frequent meals are essential to ensure that infants’ energy and nutrient needs are met.

In the United Arab Emirates (UAE) there is a high prevalence of non-communicable diseases among children, including micronutrient deficiencies, underweight and overweight; for this reason, it is necessary to promote breastfeeding and proper complementary feeding [9–11]. However, until now, most of the studies conducted on infants and young child feeding in the UAE have been confined to knowledge and practice studies on breastfeeding, while in-depth studies of complementary feeding practices are limited, revealing a clear knowledge gap [12, 13]. As healthy eating habits are established early in childhood, it is crucial to gather baseline data and assess the status using the UNICEF indicators to identify current strengths and gaps, and to monitor and measure progress over time [14]. The aim of this study was to describe the complementary feeding practices among infants and young children in Abu Dhabi, UAE and to evaluate them using the UNICEF Programming Guide: Infant and Young Child Feeding.

## Methods

### Participants and data collection

In this cross-sectional study, Emirati and non-Emirati mothers were recruited from the community (mainly university students) and from seven of the eleven maternal and child health centers that are spread across the urban, suburban and rural areas in the Abu Dhabi Capital District. The data were collected in 2017. Eligible mothers were provided with oral and written information about the study while visiting child health centers. In total, 1822 consenting mothers who met the inclusion criteria were interviewed by the research assistants using a structured questionnaire). A detailed description of the study design and sampling has been published elsewhere [15]. Prior to the main study, a pilot study was conducted using face validity in order to reduce bias. Interviews were chosen as the data collection method both to minimize misunderstandings and also to assist participants with low reading and writing skills.

### Study instrument

The anthropometric data of the children were obtained from their health cards. The questionnaire used in the study was solely developed for this specific investigation and included questions about family demographics and infant feeding practices [15]. In addition, questions about food groups, meal frequency and consumption of beverages and snack items during a typical 24-h period were also included. The findings related to the complementary feeding practices were evaluated using indicators from the UNICEF Programming Guide: Infant and Young Child Feeding [16].

According to UNICEF, the “introduction of complementary foods” is defined as “the proportion of infants aged 6-8 months who receive solid, semi-solid or soft foods”. “Minimum dietary diversity” (MDD) is defined as “the proportion of children 6-23 months of age who receive foods from 4 or more food groups” [16]. The division of food groups in this study is slightly different from the one used by UNICEF. The groups of grains, legumes and dairy products are the same in this study and in UNICEF’s specifications. However, for protein sources, fruit and vegetables some differences exist. UNICEF separates animal proteins into 2 groups consisting of eggs and other animal proteins, whereas in this study eggs are included in the animal protein group. Furthermore, in this study fruits constitute one group and vegetables another, whereas UNICEF divides fruit and vegetables into two groups based on their vitamin A content. Lastly, in this study fat is considered a separate group.

“Minimum meal frequency” (MMF) is defined as “the proportion of breastfed and non-breastfed children 6-23 months of age who receive solid, semi-solid or soft foods (but also including milk feeds for non-breastfed children) the minimum number of times or more: 2 for 6-8 months, 3 for 9-23 months, 4 for 6-23 months (if not breast fed)” [1]. The final indicator used is “minimum acceptable diet” (MAD) which is defined as “the proportion of children 6-23 months of age who had both minimum meal frequency and dietary diversity (in both breastfed and non-breastfed children)” [1, 16].

### Statistical analysis

Frequencies and proportions of diet diversity were compared to evaluate feeding between children aged < 6 months and ≥ 6 months using Chi-square tests of proportions. These groups were chosen to understand the types of food groups to which children who are introduced to complementary feeding below the recommended age of 6 months are exposed. Comparative analysis was also conducted to measure differences in main meal frequency among breastfeeding and non-breastfeeding young infants (< 6 months old), 6–8 month old breastfeeding children, 9–23 month old breastfeeding children and 6–23 month old non-breastfeeding children. These groups are based on the WHO recommendation for main meal frequency depending on age and breastfeeding status. Stratified analysis by subgroups were conducted (Tables 3 & 4) to observe trends in association. The feeding practices were compared and evaluated using the UNICEF Programming Guide: Infant and Young Child Feeding [16]. All analyses were performed using IBM SPSS Statistics Premium V.24.0, STATA v14.0 SE, and SAS 9.2 for this article.

## Results

### Sample characteristics

A total of 1578 mothers from the health centers were invited, of whom 1555 accepted. In addition, 267 mothers from convenience sampling in the community (mainly university students) agreed to participate, giving a total of 1822 mother-infant pairs participating in the study. The mean age of the mothers was 30 ± 5 years. As shown, the majority (98.1%) of the mothers were married, 75.8% had a university education and over a third (35.6%) were employed outside the home. About a third (32.7%) of the mothers were Emiratis and the rest were from various other countries] (Table 1).

**Table 1:** Family demographic and health characteristics

Characteristics	***n*** = 1822 (%)
Maternal Age (years)	
17–19	20 (1.1)
20–24	247 (13.6)
25–34	1152 (63.2)
35–51	403 (22.1)
Maternal Origin	
Emirati	596 (32.7)
Non Emirati-Arab	611 (33.5)
Non Emirati-Non Arab	615 (33.8)
Marital Status	
Married	1787 (98.1)
Un-married	35 (1.9)
Maternal Education^a^	
< High School	80 (4.4)
High School	339 (18.8)
University	1382 (76.7)
Paternal Education^b^	
< High School	39 (2.1)
High School	205 (11.3)
University	1570 (86.5)
Maternal Employment	
Employed	722 (39.6)
Not Employed	1100 (60.4)
Family Financial Well Being	
Excellent/Very Good	1211 (66.5)
Good	487 (26.7)
Fair	106 (5.8)
Poor/Very Poor	18 (1.0)

^a^21 and ^b^8 missing data

The child characteristics are shown in Table 2. At the time of the interview, almost half of the children, 46.2% (841/1822) were below 6 months of age, and 11.7% (213/1822) of the children were more than 18 months old. Most of the children (68.7%) were delivered vaginally and the mean ± SD birth weight among all children was 3073 ± 534 g.

**Table 2:** Distribution of Child Characteristics in the Study Sample

Child Characteristics	*n* = 1822 (%)
Child’s Age in Months at Interview	
< 1 (< 30 days)	23 (1.3)
1–3 (30–91 days)	399 (21.9)
4–6 (92–180 days)	419 (23.0)
7–12 (181–365 days)	472 (25.9)
13–18 (367–540 days)	296 (16.2)
19–24 (541–729 days)	213 (11.7)
Gender^a^	
Female	932 (51.4)
Male	879 (48.6)
Delivery Type^b^	
Vaginal	1252 (68.9)
Cesarean Section	566 (31.1)
Gestational Age^c^	
< 37 weeks	128 (7.0)
≥ 37 weeks	1681 (92.3)
Birth Weight (grams)	
< 37 weeks (mean ± SD)	2351 ± 727
≥37 weeks (mean ± SD)	3127 ± 424
Ever Breast Fed	
Yes	1742 (95.6)
No	80 (4.4)
Still Breast Feeding	
Yes	1186 (65.1)
No	636 (34.9)
Started Complementary Feeding	
Yes	938 (51.5)
No	884 (48.5)
Age at which Complementary Feeding was Initiated (*n* = 938)	
< 6 months (< 180 days)	261 (27.8)
≥ 6 months (≥180 days)	677 (72.2)

^a^11, ^b^4 and ^c^13 missing data

### Complementary feeding introduction

Out of the total sample of 1822 children, 938 children (51.5%) had been introduced to complementary feeding (Table 2). A total of 27.8% of the children who received complementary feeding were introduced to it at < 6 months of age, while the remaining children (72.2%) were introduced to it at 6–8 months of age, in line with the WHO recommendations of timely introduction of complementary feeding [14].

### Diet diversity

The most prevalent food groups in the diet of the 938 children receiving complementary feeding, regardless of age, were vegetables, fruits and/or grains and cereals (Table 3). Significantly more children aged **≥**6 months received animal protein and dairy products than the children aged < 6 months. One quarter, 221/871 (25.4%) of the children aged **≥**6 months had extra fats such as oils added to their food compared to 7/67 (10.4%) of the children below 6 months. The diet diversity was significantly higher among the children aged **≥**6 months than the group of younger children (Table 3). A small proportion, 14/67 (20.9%) of the children < 6 months of age, were given **≥**4 food whereas a majority of the children in this age category 50/67 (74.6%) were given 1–3 food groups and 3/67 (4.5%) were given food items other than the recommended food groups. Among the children aged **≥**6 months, 622/871 (71.4%) received at least 4 food groups as part of their complementary feeding meaning that they met the WHO recommendation for MDD, whereas 249/871 (28.6%) had lower diet diversity and hence did not meet the WHO recommendation (Table 3).

**Table 3:** Complementary feeding practices by food group and diet diversity by child’s age at interview. *N* = 938

	0–180 days (*N* = 67)		181–729 days (*N* = 871)		
	N	%	N	%	*p*-value
**Food groups**					
Animal protein	12	17.9	522	59.9	0.001
Dairy products	16	23.9	605	69.5	0.001
Pulses/legumes	8	11.9	454	52.1	0.001
Cereals/grains	47	70.1	723	83.0	0.008
Fats/oils	7	10.4	221	25.4	0.006
Vegetables	45	67.2	792	90.9	0.001
Fruits	48	71.6	739	84.8	0.001
**Diet Diversity**^**a**^					
7 food groups	3	4.5	175	20.1	0.001
6 food groups	4	6.0	164	18.8	
5 food groups	4	6.0	141	16.2	
4 food groups	3	4.5	142	16.3	
3 food groups	19	28.4	132	15.2	
2 good groups	18	24.9	62	7.1	
1 food group	13	19.4	54	6.2	

^a^A total of 4 children did not include any of the recommended food groups but ratherfood that is not recommended at this age group

These children are not included in the table

In addition to the basic recommended food groups, some children were also exposed to beverages. While a majority 40/67 (59.5%) of the children aged < 6 months who had started complementary feeding did not get any fluids other than breast milk, baby formula or water, 27/67 (40.3%) received 1–2 offerings of other beverages like juice, soda, chocolate milk or herbal tea daily. Among the children **≥**6 months of age, 43.7% (380/871) did not get fluids other than breast milk, baby formula or water, whereas 453/871 (52.0%) received 1–2 offerings of beverages like juice, soda, chocolate milk or herbal tea and 4.3% (38/871) received **≥**3 daily offerings of these beverages.

### Meal frequency

**Fig. 1:**
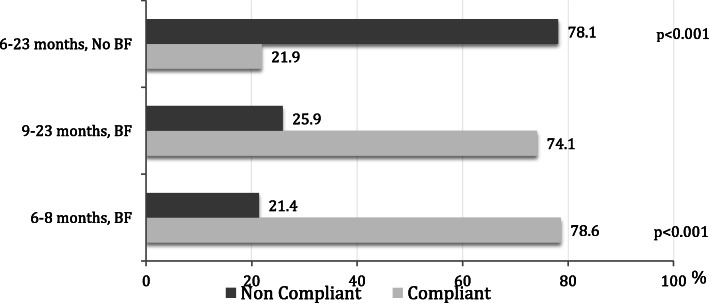
Compliance with the WHO recommendations for minimum meal frequency according to age and breastfeeding status. (*n* = 871)

Table 4 shows the meal frequency among the children according to their age and breastfeeding status for 931 children (due to 7 missing data). As shown, less than half (47.3%) of the children who were 6–23 months of age and regardless of their breastfeeding status at the time of interview were compliant with the WHO indicator for MMF. A further breakdown shows that the group of children aged 6–23 months who were not breastfeeding had a significant lower compliance as compared to the other categories of children (*p* < 0.001) (Fig. 1).

**Table 4:** Total frequencies of main meals by the child’s age and breastfeeding status. Bold numbers indicate children’s meal frequency adhering to the WHO recommendations (*N* = 931)

Child’s Age	Breast feeding status	Daily main meal frequency				Recommended daily meal frequency^**a**^
		1 meal N (%)	2 meals N (%)	3 meals N (%)	≥4 meals N (%)	
< 6 months	Yes/No	17 (27.0)	27 (42.9)	15 (23.8)	4 (6.3)	N/A
6–8 months	Yes	40 (21.4)	**88 (47.0)**	**52 (27.8)**	**7 (3.7)**	≥2
9–23 months	Yes	3 (1.3)	55 (24.6)	**131 (58.4)**	**35 (15.6)**	≥3
6–23 months	No	18 (3.9)	92 (20.1)	247 (54.0)	**100 (21.9)**	≥4

^a^World Health Organization. Infant and young child feeding a tool for assessing national practices, policies and programmes. WHO, Geneva 2003

Apart from the main meals some children also received snacks primarily consisting of fruits, biscuits, cake, natural or sweetened yoghurt, chips, vegetables, chocolates, nuggets and/or baby porridge. Among the children aged < 6 months and ≥ 6 months, 43/67 (64.2%) and 733/871 (84.2%) received snacks, respectively. Snacks were given once daily to 27/67 (40.3%) of the children who were <  6 months of age as compared to 209/871 (24.2%) of those who were **≥** 6 months of age. Two snacks or more were given to 16/67 (23.9%) and 524/871 (60.2%) to the children < 6 months and **≥** 6 months of age, respectively.

### Minimum acceptable diet (MAD)

Among the 938 children receiving complementary feeding, 871 were **≥** 6 months of age and 622 of these children received at least 4 food groups as part and hence met the WHO recommendation for MDD. Furthermore, 409 of the 871 children received the MMF according to their age and breastfeeding status. MAD, as defined by the proportion of children 6–23 months of age who had both minimum meal frequency and dietary diversity, were met by 36.2% (316/871) of the children.

## Discussion

As one of the first studies to investigate complementary feeding practices in the UAE using the UNICEF indicators, this study revealed several interesting results. Although most of the children were introduced to complementary feeding in a timely manner, more than a quarter were introduced too early. A majority of the children aged **≥**6 months received at least four of the recommended basic food groups but, alongside that, many of them had also already been introduced to sweetened foods, primarily in the form of snacks. Most children who were still breastfeeding met the recommendation for MMF, whereas only a quarter of the non-breastfeeding children met this recommendation. Overall, an MAD was found in only around a third of the children, raising concerns about the current feeding practices.

Most mothers (72.2%) had followed the WHO recommendations of timely introduction of complementary feeding at 6–8 months of age, unlike in another recent study in the UAE where 83.5% of the children had been exposed to solid food before the age of 6 months [12]. As socio-demographic factors have been shown to affect feeding practices, the difference may be explained by the multi-national composition of the sample in the current study and the mothers’ considerably higher level of education [12, 17]. Unlike in studies from Ethiopia, late introduction of complementary feeding was not identified as a problem in this study [18]. This difference may be related to differences in food security and accessibility. Although most countries have endorsed the WHO recommendations, different guidelines can be found in different geographical regions, often depending on the level of food security and the relevant country’s level of development [19–22]. Some guidelines recommend introducing complementary feeding at 4–6 months of age [7, 19]. In Ireland, following the ESPGHAN guidelines, three quarters of the infants adhered to these recommendations and in a national sample from the US, around 40% of the infants were doing the same [7, 19, 23, 24]. All feeding guidelines advise against the introduction of complementary feeding prior to 4 months of age, as it has been associated with major health risks [1, 7, 19]. In the current study around a quarter of the children were introduced to complementary feeding at < 6 months of age, but as the exact age of introduction is unknown it is not possible to evaluate the extent of the potential associated risks.

As expected, the MDD was better among the older children, which is most likely associated with the children having had more time to be exposed to a wider variety of food items, tastes and textures. According to the WHO a variety of animal proteins should be introduced from 6 months of age as a way of preventing iron deficiency [25]. In this study, only 6 out of 10 children aged **≥**6 months were given animal protein and only a small proportion had fats or oil added to their food, despite infants’ higher protein and fat requirements compared to older children and adults [26]. In this study many of the children aged > 6 months had an acceptable MDD. This is on par with countries like Peru and Colombia, but is higher than other Middle Eastern countries such as Egypt, Jordan, Iraq and Palestine where the percentage varies between 34.7 and 50.3% [27–29].

Although infants have an inborn preference for sweet tastes, repeated exposure to new foods increases the acceptance of other flavors and hence the development of new preferences [30]. On the other hand, if infants are exposed from an early age to energy-dense food with a high sugar content it may reduce their intake of nutrient-dense food, negatively affecting their growth [31]. Furthermore, early exposure to sugar-containing food in infancy has also been associated with a higher incidence of dental caries among preschoolers [32]. In the current study many infants were exposed to sugar-containing food from a very young age. Although these findings stand in strong contrast to the recommendations, others have reported similar results [32]. It is possible that the cumulative effect of a low intake of animal protein and an early exposure to sweet food and beverages is associated with the high prevalence of iron deficiency, abnormal growth and dental caries that has been reported among young children in the UAE [33, 34].

Although a majority of the children who were still breastfeeding met the recommended MMF, only 21.9% of the non-breastfeeding children aged > 6 months were receiving the recommended minimum of at least 4 main meals per day. These results are considerably lower than those in most of the 135 countries recently surveyed by UNICEF [29]. It is also worth noticing that the high MDD and high MMF that were reported among some of the children in this study aged < 6 months are not compliant with the IYCF guidelines and may be hazardous due to the risk of choking, indigestion and food intolerance [1].

An MAD was found among 36.2% of the children in this study. This is on par, or higher, compared to other Middle Eastern countries: 33.3% in Jordan, 23.3% in Egypt and 15.4% in Yemen [29]. However, compared to many other countries with similarly high food security levels as the UAE, the MAD compliance in this study is considerably lower [29].

As in all research, this study has its strengths and limitations. The UAE is a country with a highly diverse population consisting of 11.48% UAE nationals and 88.52% non-UAE nationals, many of whom live in the country for a significant time period [35]. It is therefore a strength that the study included both UAE nationals and non-UAE nationals residing in different geographical areas. Another strength is the large sample from the community. The data collection was not confined only to the recommended food groups but also included open-ended questions about snack items and beverages, which could be considered as another strength as it provides a comprehensive picture of the children’s overall intake.

As for limitations, recall bias commonly seen in cross-sectional studies, may also be an issue in this study. Not using exactly the same food groups as UNICEF could be seen as a limitation when evaluating diet diversity, but since vitamin A deficiency is not a major health problem among infants in the UAE the grouping into fruits and vegetables should be sufficient to capture children’s intake of vitamins and minerals [36]. Efforts were made to include a representative sample of mothers with young children. The big study sample and the inclusion of participants from different demographic backgrounds, geographic locations and levels of urbanization means that the results may be generalizable to the emirate of Abu Dhabi and other similar emirates. However, the results may not be generalizable to emirates with a different population demography and level of economic development, and this can also be seen as a limitation.

## Conclusion

In conclusion, even though a majority (72.2%) of the children were introduced to complementary feeding in a timely manner, and a majority (71.4%) of them had an acceptable MDD, only a quarter of the children who had discontinued breastfeeding had an acceptable MMF. Furthermore, many of these were given sweetened snacks and beverages. Only 36.2% of the children aged ≥6 months had an MAD; given that the study was conducted in the UAE, an economically developed country with high food security, this seems likely to be due to poor knowledge rather than food availability problems [37]. The adoption of unhealthy food habits from infancy is posing a threat to children’s overall health, both short-term and long-term.

The UAE has adopted the WHO infant feeding policies and previously made national infant and young child feeding policies available to the public [38]. Despite efforts, we have not been able to track down any current national policies. Hence, our recommendation moving forward is that the policies should be revised and again made available to the public to ensure that they can be used, and recommendations can be enforced, to improve the feeding status of infants and young children. This should include implementing effective health education programs including professional training, and community-based approaches initiatives to give families the appropriate knowledge, support and encouragement to adopt better food practices at home.

## Supplementary information

**Additional file 1.** Questionnaire. The questionnaire used in the study was developed for this specific investigation to obtain information about family demographics and infant feeding practices.

## Data Availability

The datasets generated and/or analysed during the current study are not publicly available mostly due to Departmental /institutional policies but may be available from the corresponding author on reasonable request.

## Abbreviations

BMI: Body mass index
CF: Complementary feeding
ESPGHAN: European Society for Pediatric Gastroenterology, Hepatology and Nutrition
IYCF: Infant and young child feeding
MAD: Minimum acceptable diet
MDD: Minimum dietary diversity
MMF: Minimum meal frequency
SD: Standard deviation
UAE: United Arab Emirates
UNICEF: United Nations Children’s Fund
WHO: World Health Organization
