# Supplementary material for: Complementary feeding practices among infants and young children in Abu Dhabi, United Arab Emirates
Source: BMC Public Health. 2020 Aug 27;20:1308. doi: 10.1186/s12889-020-09393-y (PMC7453515; doi:10.1186/s12889-020-09393-y)
Supplement: Supplementary file 1 — Additional file 1. Questionnaire. The questionnaire used in the study was developed for this specific investigation to obtain information about family demographics and infant feeding practices. [file 12889_2020_9393_MOESM1_ESM.doc]

**ID:**

**_ _ _**

| **Serial No.** |  | **Date of interview** | (dd/mm/yyyy) _____/_____/__________ |
| --- | --- | --- | --- |
| **Name of this Child** |  | **Site Code** |  |
| **Name of University** |  | **Name of Hospital where this child was delivered** |  |
| **Name of Interviewer** |  | **Mobile:** |  |

**All Information collected will generate group statistics only. Your answers are voluntary, confidential and anonymous.**

|  | **Question** | **Answer** |
| --- | --- | --- |
| **PART I : DEMOGRAPHIC AND HEALTH DATA FOR THE PARENTS AND THE CHILD (PLEASE CIRCLE YOUR ANSWER**) | | |
|  | **MOTHER’S INFORMATION** |  |
|  | How old are you?  Your date of birth (dd/mm/yyyy) _____/_____/__________ | ___ ___ years old |
|  | Marital status | 1. Married 2. Divorced 3. Widowed |
|  | What is your nationality? | 1. UAE 2. Other Arab Nationality 3. Asian 4. Other |
|  | How would you describe yourself? **(Check all that apply)** | 1. Student _______ 2. Employed _______ 3. Housewife _______ 4. Other (specify) _______ |
|  | If you are a student in the university, which of the following applies to you to your current academic level? | 1. ABP_____________ 2. General ED__________ 3. University Major (Specify)____________________ |
|  | If you are an employee at the University, what is the highest level of education have you completed | 1. Below High School 2. High School 3. Some College 4. Finished College 5. University or Higher (Masters, Doctorate, etc) |
|  | What is the educational level of the father of this baby (name)? | 1. No education 2. Reads & writes 3. Primary 4. High school 5. College 6. University or higher |
|  | How tall are you without shoes | --------- Feet --------- inches (e.g. 5 ft 10 inches) OR  _______ cms (e.g. 175 cms) |
|  | How much do you weigh now? | ____. ____ pounds (e.g. 110.5 pounds or 110 pounds 8 ounces) OR  _____.______ kgs (e.g. 45.7 kgs) |
|  | How much did you weigh **before you became pregnant** with this baby (name)? | ____. ____ pounds (e.g. 110.5 pounds or 110 pounds 8 ounces) OR  _____.______ kgs (e.g. 45.7 kgs) |
|  | Can you tell how much you weighed immediately after you delivered this baby (name)? | ____. ____ pounds (e.g. 110.5 pounds or 110 pounds 8 ounces) OR  _____.______ kgs (e.g. 45.7 kgs) |
|  | What is the monthly income in AED in your family? | ___________________ Dhirams  Don’t know ____________ |
|  | How many people in your house does this income support? | ______________________ |
|  | Considering your monthly family income, how would you rate your and your family’s overall financial well-being | 1. Excellent 2. Very Good 3. Good 4. Fair 5. Poor 6. Very Poor |
|  | How many children of your own do you have including this child (name)? | ___________ (No. of children. Use 1 if this child (name) is the only child) |
|  | What is the gender (name) of this child (name)? | 1. Male 2. Female |
|  | What is the birth order of this child (name)? | 1. First 2. Second 3. Third 4. Fourth 5. Fifth or more |
|  | How much did this child (name) weigh at birth? | ______ pounds _____ ounce OR  ______ grams |
|  | What was the length of this child (name) at birth | ________ centimeters OR  ________ inches |
|  | What was the gestational age of this child (name) at birth: | ________ weeks _________ days |
|  | How much does this child (name) weigh NOW? | ______ pounds _____ ounce OR  _______ kilograms ______ grams |
|  | How was this child (name) delivered? | 1. Vaginal 2. Planned Cesarean 3. Acute/Induced Cesarean |
|  | What is the birth date of this child (name)? | (dd/mm/yyyy) _____/_____/__________ |
|  | How many children who live in your house (include step children and other’s children) including this child (name) are below 18 years old? | ______________ |
| **PART IIA : CHILD FEEDING KNOWLEDGE, ATTITUDES AND PRACTICE AMONG MOTHERS** | | |
|  | Question | Answer |
|  | Have you ever breastfed this child (name)?  *(“Ever” means if mother has breast fed at all to this baby)* | 1. Yes 2. No **(Skip to Q30)** |
|  | Are you breastfeeding this child (name) now? | 1. Yes 2. No **(Skip to Q28)** |
|  | Are you exclusively breastfeeding (no water or liquid) this child now? | 1. Yes 2. No **(Skip to Q31)** |
|  | **For mothers who answered “Yes” to Q25 AND “No” to Q26**. How old was this child (name) when you stopped **exclusively breastfeeding (only mother’s milk)** this child? | ____________ in months ________ days |
|  | **For mothers who answered “Yes” to Q25 AND “No” to Q26**. How old was this child (name) when you stopped **giving any breast milk** to this child? | ____________ in months ________ days |
|  | **For mothers who answered “No” to BOTH Q25 AND Q26**  What are you feeding your child now? | 1. Formula feeding only   2) Feeding with formula and solid & liquid food   1. Feeding with solid and liquid food – No formula |
|  | **For mothers who answered** “No” to Q27  What are you feeding your child now? | 1. Breast milk and formula 2. Formula feeding only 3. Breast milk, formula and other solid & liquid food 4. Feeding with formula and solid & liquid food 5. Feeding with solid and liquid food – No formula |
|  | **For those who answered Yes to Q25 AND Q26:**  How soon did you start breastfeeding after delivery of this child (name)? | 1. In less than an hour after delivery 2. About an hour or so after delivery 3. 1-2 hours after delivery 4. 2.5 – 5 hours after delivery 5. 5-10 hours after delivery 6. >10 hours after delivery 7. Started breastfeeding at home after discharge from hospital |
|  | Did you ever think of breastfeeding this child (name) when you were pregnant with this child (name)? | 1. No 2. Yes |
|  | **If “Yes” to Q33,** which month of your pregnancy did you start thinking that you may breastfeed this child (name)? | 1. First 3 months 2. 4-6 months 3. 7 months or later 4. Don’t remember |
|  | Do you get support and encouragement from family members and relatives on breastfeeding | 1. No 2. Yes |
|  | **If “Yes” to Q35,** who are the people in your family who support and encourage you to breastfeeding this child (**Check all that apply)** | 1. Mother-in Law 2. Mother 3. Husband 4. Other relatives (specify)___________________ 5. Other non-relatives (specify) _______________ |
|  | Did your child (name) stay with you in the same room during your hospital stay (Rooming-in) | 1. No 2. Yes |
|  | To the best of your knowledge, for how long should a mother breastfeed her baby? | 1. Don’t have to breastfeed 2. Less than 6 months 3. 6 to 12 months 4. 13 to 18 months 5. 18 to 24 months 6. 24 months 7. More than 24 months 8. Don’t know |
|  | To the best of your knowledge, at what age (of the child) should a mother start giving solid food? | 1. Less than 6 months 2. 6 months 3. More than 6 months 4. I don’t know |
|  | Where do you usually get information about breastfeeding? (**Check all that apply**) | 1. Hospital 2. Health Center 3. Private Clinic 4. Family members 5. Other Relatives 6. Friends and Non-Relatives 7. Television/Radio 8. Social Media 9. Others (specify) |
|  | While you were pregnant with this child, did someone discuss and/or advise to you about breastfeeding? | 1. No 2. Yes |
| 42 | If Yes to Q41, then please name the person(s) who discussed and/or advised to you on positive or negative things about breastfeeding during your pregnancy with this child. **Check all that apply.** | Discussed/Advised  Positive Negative   1. My Obstetrician/Nurse/Health Professional   at the hospital/Clinic ____ ____   1. A Lactation Specialist at the hospital/Clinic ____ ____ 2. My Mother ____ ____ 3. My in-Laws (includes mother in-law) ____ ____ 4. My Husband ____ ____ 5. Other Relatives ____ ____ 6. Friends and other non-relatives ____ ____ 7. Others (Specify)_________________ ____ ____ |
| 43 | After you delivered this child, did someone discuss and/or advise to you about breastfeeding? | 1. No 2. Yes |
| 44 | If Yes to Q43, then please name the person(s) who discussed and/or advised to you on positive or negative things about breastfeeding after you delivered this child. **Check all that apply.** | Discussed/Advised  Positive Negative   1. My Obstetrician/Nurse/Health Professional   at the hospital/Clinic ____ ____   1. A Lactation Specialist at the hospital/Clinic ____ ____ 2. My Mother ____ ____ 3. My in-Laws (includes mother in-law) ____ ____ 4. My Husband ____ ____ 5. Other Relatives ____ ____ 6. Friends and other non-relatives ____ ____ 7. Others (Specify)_________________ ____ ____ |
|  | **PART IIB. COMPLEMENTARY FEEDING (ASK ONLY TO MOTHERS WHO HAVE STOPPED EXCLUSIVELY BREASTFEEDING THIS CHILD)**  **RESPONSES 2 or 3 to Q30 OR responses 3 or 4 or 5 to Q31** | |
| 45 | At what age did you start giving your child water? | 1. Less than 1 month 2. 1 month 3. 2 months 4. 3 months 5. 4 months 6. 5 months 7. 6 months 8. More than 6 months |
| 46 | What was the age of your child when you started complementary foods (CF) such as cereal, meat, fish, yoghurt, egg, cheese, fruits and vegetables etc? | 1. Less than 6 months 2. 6 months 3. More than 6 months 4. Formula feed only 5. I don’t know |
| 47 | How many times do you give your child Complementary Food? | ________ times |
| 48 | How many times do you give your child snacks? | ________ times |
| 49 | What food(s) have you been giving your baby as complementary food (CF)? **Check all that apply** | 1. animal proteins (for example: meat, fish, chicken, liver, egg..etc.) 2. 2. milk proteins (for example: yoghurt, cheese ….etc.) 3. plant proteins (pulses, lentils and legumes) 4. cereals 5. vegetables (for example: red, dark green or orange) or 6. fruits (red or orange fruits, dates) 7. chocolates and sweets 8. chips and other fried food 9. other (specify)__________________________________________ |
| 50 | How many times do you give your child Complementary Food (CF) in a day? | ________ times |
| 51 | What do you typically include in a main meal for this child? **(Open ended, Will code them during data input)** | _________________ |
| 52 | How much CF do you give to your child at each meal?  (show a 250 ml bowel or cup) | 1\2 of a 250 ml cup  2/3 of a 250 ml cup  3/4 of a 250 ml cup  A full 250 ml cup  Others ( specify) |
| 53 | Are the CF that you give your child, ready-made from the market (eg. Cerelac or other canned baby food), home made, or both | 1. Ready-made (Available in the market) 2. Home made 3. Both ready-made and home made |
| 54 | If Ready-made of both, how do you prepare that ready-made CF? | 1. Following the instructions written on the container 2. Without following the instructions written on the container |
| 55 | What type of food do you give to your child as a snack? (**Open ended, Will code them during data input)** | _________________ |
| 56 | How many times do you give your child snacks in a day? | ________ times |
| 57 | What type of drinks do you give to your child as a snack? **(Open ended, Will code them during data input)** | _________________ |
| 58 | How many times do you give your child drinks in a day? | ________ times |

| **PART III: REASONS FOR NOT INITIATING OR CONTINUING TO BREASTFEEDING**  **If you exclusively breastfed your infant for <6 months**, **or stopped breastfeeding at any age of the child, or never breastfed this child at all,**  what barriers prevented you from breastfeeding? **CHECK ALL THAT APPLY** | | | | |
| --- | --- | --- | --- | --- |
|  |  | **REASONS** | | |
|  |  | **For those who breastfed exclusively for Less than SIX Months** | **For those had stopped ANY breastfeeding after Initiation** | **For those who NEVER Initiated Breastfeeding** |
|  | **Variables** |  |  |  |
| 59 | Not enough breast milk production |  |  |  |
| 60 | Concern that baby will not receive adequate nutrition from mother’s milk |  |  |  |
| 61 | Concern that baby is too small to breastfeed |  |  |  |
| 62 | Concern that certain food mother eat will make the baby sick if breastfed |  |  |  |
| 63 | Concern that breastfeeding is ruining the mother’s beauty |  |  |  |
| 64 | Breastfeeding is always stressful |  |  |  |
| 65 | Breastfeeding takes a lot of time |  |  |  |
| 66 | Concern that breastfed babies are smaller compared to formula fed babies |  |  |  |
| 67 | Concern about going back to work |  |  |  |
| 68 | Concern about going back to school |  |  |  |
| 69 | Embarrassment of breastfeeding in public places |  |  |  |
| 70 | Sore or painful nipples |  |  |  |
| 71 | No breast pump available to express breast milk |  |  |  |
| 72 | Poor latching on |  |  |  |
| 73 | Working in evening or night shifts |  |  |  |
| 74 | Concern that medications used by mother are harmful to the baby |  |  |  |
| 75 | No breastfeeding experience |  |  |  |
| 76 | Tried but failed during previous breastfeeding experience |  |  |  |
| 77 | Lack of support from hospital staff when asked to help with breastfeeding |  |  |  |
| 78 | Lack of information from the practitioners at the clinic about the benefits of exclusive breastfeeding |  |  |  |
| 79 | Lack of support from the Mother-in-Law |  |  |  |
| 80 | Lack of Support from Husband |  |  |  |
| 81 | Lack of support from relatives |  |  |  |
| 82 | No rooms or private space for breastfeeding or expressing breast milk |  |  |  |
| 83 | Lack of information about whom to contact for help when faced with challenges with breastfeeding |  |  |  |
| 84 | Baby gets hospitalized frequently |  |  |  |
| 85 | Doctor’s advice to mother not to breastfeed due to illness |  |  |  |
| 86 | Do not see any benefits of breastfeeding |  |  |  |
| 87 | Don’t know much about breastfeeding |  |  |  |
| 88 | Other reasons (Specify) |  |  |  |
